# Metal–Organic Framework (MOF)-Derived Metal Oxides for Selective Catalytic Reduction (SCR) of NO_x_

**DOI:** 10.3390/molecules30132836

**Published:** 2025-07-02

**Authors:** Yu Zhang, Rui Wang

**Affiliations:** School of Environmental Science and Engineering, Shandong University, Qingdao 266237, China

**Keywords:** MOF-derived metal oxides, selective catalytic reduction, NO_x_, catalytic performance

## Abstract

Metal–organic frameworks (MOFs) are a novel type of porous crystalline materials assembled from metal ions and organic linkers. Their derivatives can inherit characteristics such as high specific surface area, tunable porosity, and unique topological structures, which make MOF-derived metal oxides ideal catalysts for the selective catalytic reduction (SCR) of NO_x_. This review focuses on the synthetic strategies of MOF-derived metal oxides and the latest progress of oxides derived from various typical MOFs materials (including MILs, ZIFs, UiO, BTC series, MOF-74, MOF-5, and Prussian blue analogs, etc.) in the catalytic reduction in NO_x_, and analyzes the mechanisms for the enhanced catalytic performance. In addition, the challenges and prospects of MOF derivatives in catalytic applications are discussed. It is hoped that this review will help researchers understand the latest research progress of MOF-derived metal oxide materials in the catalytic removal of NO_x_ pollution.

## 1. Introduction

With the increasing global emphasis on environmental protection, controlling air pollution has become a significant challenge faced by countries around the world. Among various air pollutants, nitrogen oxides (NO_x_) have attracted considerable attention due to their multiple environmental hazards [1]. NO_x_ not only exacerbates global warming and depletes the ozone layer but also leads to acid rain and photochemical pollution, making it a key target in air pollution control [2,3]. These harmful gases are primarily generated from power plants, industrial production facilities, and vehicle exhaust emissions [4,5]. Despite the rapid development of clean energy, fossil fuels still dominate at present [6]. Coupled with the economic growth-driven industrial expansion and the surge in the number of vehicles, the emissions of NO_x_ are expected to rise rapidly in the short term. As public environmental awareness grows and environmental standards become stricter, the efficient purification of NO_x_ from industrial exhaust gases has become an urgent issue. Among various denitrification technologies, selective catalytic reduction (SCR) stands out for its excellent purification effect and environmentally friendly characteristics [7]. This method, by injecting reducing agents such as ammonia (NH_3_) [8,9], carbon monoxide (CO) [10,11], hydrocarbons (HC) [12,13], or hydrogen (H_2_) [14,15], can effectively achieve the clean conversion of NO_x_.

In the field of denitrification catalysis, metal oxide materials have been attracting much attention [16,17]. Vanadium-based catalysts represented by V_2_O_5_-WO_3_/TiO_2_ (VWTi) have demonstrated excellent denitrification efficiency in the temperature interval of 300–400 °C and have been commercially applied, but their low-temperature catalytic performance still needs to be improved [18]. In order to meet the demand for emission reduction in high energy-consuming industries such as steel, cement, and glass, the development of SCR catalysts that can operate efficiently in the low-temperature range of 50–250 °C is particularly important [19]. Studies have shown that transition metal oxides such as Mn, Ce, Co, and Fe have significant catalytic activity at low temperatures [20]. However, traditional preparation processes (e.g., solvothermal, co-precipitation, and solid-phase methods) often struggle to precisely regulate the material structure, and the resulting metal oxides usually suffer from uneven morphology, narrow pore size, and low porosity. These structural defects easily lead to poor dispersion and easy agglomeration of the active components, thus reducing the accessibility of the catalytic sites and affecting the overall catalytic performance.

In the last two decades, metal–organic frameworks (MOFs) have garnered increasing attention as a unique class of two- or three-dimensional crystalline materials [21]. The materials are built from various metal nodes and organic ligands relying on various interactions, such as hydrogen bonding, π-π interactions, van der Waals forces, and other bonding mechanisms [22]. The structural library of MOFs has been greatly enriched due to the large number of combinations of various building units [23]. In recent years, researchers have successfully developed many MOF materials, including ZIF-8, ZIF-67, MIL-53, MIL-100, MIL-125, MOF-74, MOF-5, UiO-66, HKUST-1, etc. [24,25,26]. MOFs possess unique porous structures, tunable functionalities, high crystallinity, large specific surface areas, customizable functional groups, and structural flexibility, making them highly attractive for applications such as denitrification catalysts [27]. For instance, MOFs exhibit an exceptionally high surface area of up to 10000 m^2^/g, far exceeding that of zeolites and activated carbon. However, insufficient activity, low yield, and poor chemical stability are the main bottlenecks of MOFs in denitrification. The study of metal oxide catalysts derived from MOFs brings hope for solving these problems. MOF derivatives can be obtained by high-temperature treatment or chemical reaction. During these processes, metal ions in the MOFs structure may be partially or completely released from the framework, leading to structural changes and the formation of derivatives [28]. These MOF derivatives retain some of the excellent properties of MOFs, such as high specific surface area, porous structure, and unique topological structures [29,30,31]. Compared with MOFs, MOF derivatives exhibit better thermal and chemical stability [32]. Moreover, compared with metal oxides prepared by traditional methods, MOF derivatives can provide more exposed and uniformly distributed active sites, thereby demonstrating superior catalytic performance. In addition, the composition, microstructure, and macroscopic morphology of MOF-derived metal oxides can be tuned by precisely selecting the type of metal precursor, the structure of the MOF template, and the synthesis process parameters [33]. This highly customizable synthesis strategy provides an effective way for the targeted optimization of material properties.

Although significant progress has been made in the field of NO_x_ removal using MOFs and their derived materials, there remains a lack of systematic reviews specifically focusing on the application of MOF-derived metal oxides in selective catalytic reduction (SCR) denitrification. Therefore, this review concentrates on this crucial area, summarizing recent advances in the design of MOF-derived metal oxide catalysts for denitrification, with particular emphasis on the synthesis techniques of MOF-derived catalysts and their current research status in NO_x_ selective catalytic reduction. Additionally, it provides a critical discussion on the challenges faced by MOF derivatives, as well as their future development directions. We believe this high-quality review will inspire more research teams to develop highly efficient denitrification catalysts, thereby advancing progress in environmental remediation efforts.

## 2. Preparation Method of MOF-Derived Metal Oxides

MOF derivatization synthesis is a relatively simple preparation method, which can conveniently obtain porous structural materials with controllable morphology, size, crystalline phase, and physicochemical properties [34]. Compared with traditional chemical and physical preparation processes, the preparation of metal oxides using MOFs as precursors has unique advantages. MOF materials themselves have a coordination network structure composed of metal ions and organic ligands. When subjected to programmed temperature pyrolysis in a specific atmosphere (air or inert gas), the organic components gradually decompose and release small-molecule gases (such as CO_2_, NO_2_, etc.), eventually transforming into porous metal oxides. By precisely controlling the pyrolysis parameters (temperature, time), the pore characteristics of the MOF precursors can be effectively inherited, achieving synergistic control of the product’s high specific surface area and well-developed pore structure [35]. More importantly, by simply adjusting the annealing time and temperature, the key parameters of the derived oxides, such as chemical composition, pore size, and surface properties, can be precisely controlled. So far, the preparation of metal oxides using MOFs as a sacrificial template mainly includes self-pyrolysis, loading self-pyrolysis, and chemical reaction (Table 1). To date, various metal oxides have been derived from MOF precursors, including MnO_x_ [36], Fe_2_O_3_ [37], ZnO [38], NiCo_2_O_4_ [39], Co_3_O_4_/LaCoO_3_ [40], Bi_2_O_3_/CeO_2_ [41], etc.

### 2.1. Self-Pyrolysis

**Figure 1:**
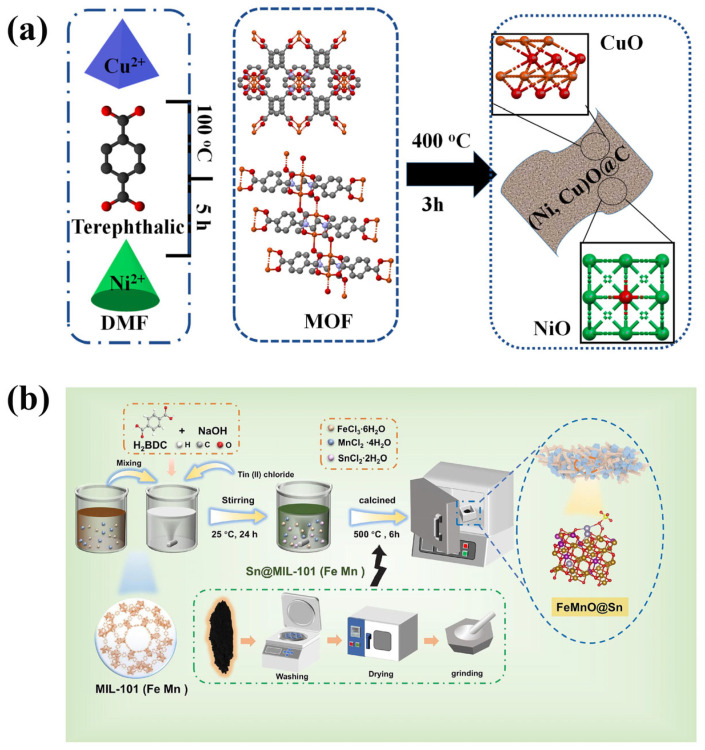
Schematic representation of the materials preparations (**a**) (Ni, Cu)O@C [42]; (**b**) FeMnO@Sn catalysts [43].

The self-pyrolysis method uses MOFs as templates without adding any additives. The morphology and specific surface area of the prepared metal oxides are inherited from those of the MOFs. Alhakemy et al. [42] synthesized terephthalate metal–organic frameworks (MOFs) using a surfactant-assisted solvothermal method (Figure 1a). Specifically, a certain amount of metal salt (Cu(NO_3_)_2_, NiCl_2_) and cetyltrimethylammonium bromide (CTAB) were dissolved in a mixture of N,N-dimethylformamide (DMF) and acetonitrile (ACN), which was then combined with a DMF solution containing terephthalic acid and CTAB. After stirring the mixture at 100 °C for 5 h, a blue precipitate was filtered out, washed with DMF, and dried in an oven at 100 °C to obtain the MOF precursor. Finally, the precursor was heated to 400 °C at a rate of 10 °C/min and maintained at this temperature for 3 h in air to yield the target metal oxide. A research team from Chongqing University has successfully developed a magnetic trimetallic MOF-derived Fe-Mn-Sn oxide heterostructure (FeMnO@Sn), with adjustable morphology, size, and Sn content. The preparation process is shown in Figure 1b. It was observed that all ternary oxide samples exhibited a unique macroscopic morphology resembling a “bird’s nest”. As the amount of added SnCl_2_ increased gradually, the morphology of the samples evolved from rod-shaped to sheet-on-rod particles, and eventually to irregular particles. During this process, the introduction of a small amount of Sn played a key role in controlling the size and shape of the samples. However, as the Sn content continued to increase, the tendency for particle agglomeration also increased, while the density decreased and the degree of structural damage increased [43]. In Chen et al.’s study, two-dimensional MOF nanosheets formed by coordination self-assembly of magnesium ions and 1,4-benedicarboxylic acid (BDC) were synthesized by a simple bottom-up one-pot solvothermal synthesis method using PVP as the structure guide agent. Through the subsequent one-step self-template pyrolysis process, omasum-like MOF-derived porous nanosheets were obtained, with MgO/C heterostructures uniformly distributed on the surface, effectively avoiding the problem of random pore plugging [44]. An innovative one-step calcination method for the conversion of the Prussian blue analog (PBA) to Mn_2_O_3_-Fe_3_O_4_ was used by Wang et al. The hollow porous nanocubic structure of PBA was largely preserved compared to most conventional processes [45]. Huang et al. [46] prepared a mesoporous ternary metal FeCoNi oxides (FCN-MOS) by a one-step hydrothermal method followed by calcination, starting from the Fe-MIL-88B precursor. The FCN-MOS is mainly composed of a ternary phase composite of α-Fe_2_O_3_, CoFe_2_O_4_, and NiFe_2_O_4_. SEM analysis revealed that the derivative successfully retained the hexagonal rod-like morphology of the parent MOFs. Jiang et al. [47] formed composite metal oxides characterized by strong intermetallic interactions, high specific surface area, and small-sized nanoparticles by pyrolysis of MnCe-MOF in an inert atmosphere, followed by pyrolysis of MnCe-MOF in an air atmosphere. Bi et al. [48] Synthesis of Pd@ZrO_2_ catalysts with strong interfacial interactions using in situ grown Zr-based metal–organic framework (MOF) Pd@UiO-66 as precursor.

### 2.2. Loading Self-Pyrolysis

**Figure 2:**
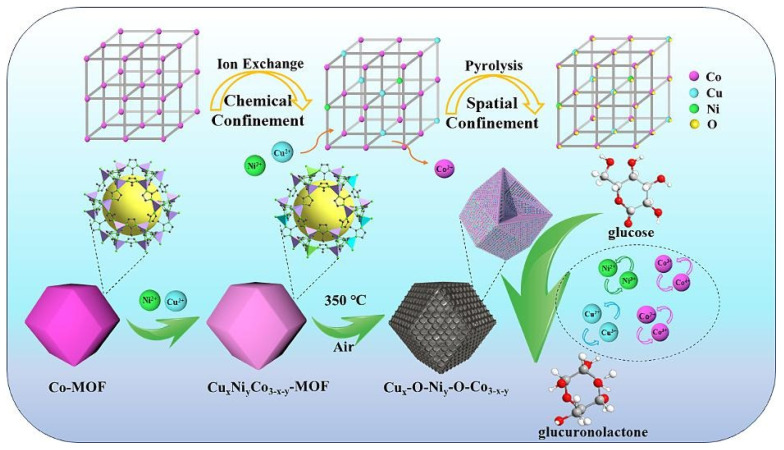
Schematic diagram of synthesis mechanism of Cu_x_-O-Ni_y_-O-Co_3−x−y_ [49].

Zhang et al. [49] introduced Cu^2+^ and Ni^2+^ into Co-MOF via ion exchange under chemical confinement regulation and then transformed the trimetallic MOF into the composite oxide Cu_x_-O-Ni_y_-O-Co_3−x−y_ through pyrolysis. SEM results showed that the derived oxide retained the dodecahedral shape of the MOF precursor but exhibited slightly wrinkled surfaces. The detailed mechanism is as follows (Figure 2): when Co-MOF was immersed in a solution of Cu(NO_3_)_2_·3H_2_O and Ni(NO_3_)_2_·6H_2_O, the Co-N coordination bonds in Co-MOF, constructed by Co^2+^ and 2-methylimidazole, were etched by H^+^ produced from the hydrolysis of Cu^2+^ and Ni^2+^, causing some Co-N bonds to break. The released Co^2+^ was confined near the etching sites due to the highly ordered framework structure of Co-MOF. Given the similar ionic radii and preferred coordination configurations of Cu^2+^; Ni^2+^; and Co^2+^, Cu^2+^, and Ni^2+^ could replace the released Co^2+^ to form new coordination bonds with the nitrogen atoms of 2-methylimidazole, successfully confining Cu^2+^ and Ni^2+^ at the lattice nodes of Co-MOF and thus achieving the transformation from Co-MOF to Cu_x_Ni_y_Co_3−x−y_ -MOF without altering the crystal structure of Co-MOF. Subsequently, Cu_x_Ni_y_Co_3−x−y_ -MOF was calcined in air at 350 °C for 2 h to produce Cu_x_-O-Ni_y_-O-Co_3−x−y_. During this process, the rigid framework of Co-MOF orderly fixed the metal ions of Co^2+^, Cu^2+^, and Ni^2+^, preventing their agglomeration or leaching. Meanwhile, the metal ions were rearranged within the confined space to form metal oxides with specific crystal structures, retaining the morphology of the parent Co-MOF. Additionally, the organic ligand decomposed due to its poor thermal stability, generating gases such as CO_2_, H_2_O, and NO_x_. Zhu et al. [50] synthesized a series of rough and porous cubic CeO_2−x_-MnO_x_ catalysts using manganese nitrate impregnated cerium metal–organic frameworks as precursors after a conventional calcination step. The study revealed that the CO gas released during the thermal decomposition of the MOFs played a crucial role in modulating the valence states of Ce and Mn. Specifically, it significantly increased the relative concentrations of Ce^3+^ and Mn^3+^ in the CeO_2−x_-MnO_x_-2.5 sample, thereby promoting the formation of oxygen vacancies. Shokry et al. [51] coated Cu-BTC MOFs on MnO_2_ nanorods through simple layer-by-layer assembly, and then generated MnO/Cu-C catalysts through high-temperature reduction. The research team from Xi’an Jiaotong University used the calcination method to synthesize the CoCeO_x_ bimetallic catalyst by partially replacing Ce in Ce-UiO-66 with Co. It was found that the Co that entered the Ce-UiO-66 framework exerted a limiting effect on the Ce cations, thereby interfering with the growth process and the crystallinity of the Ce-UiO-66 structure to a certain extent. After the pyrolysis treatment, the resulting CoCeO_x_-M bimetallic oxide basically retained the octahedral structure, with a specific surface area as high as 68.2 m^2^/g, which is significantly higher than that of the bulk composite oxide CoCeO_x_-B prepared by the traditional co-precipitation method (with a specific surface area of only 13.6 m^2^/g) [52]. Zou and colleagues immersed a pre-synthesized oxalate-based MOF precursor into a 1 mg/mL RuCl_3_·3H_2_O aqueous solution. After thorough soaking and drying, the material was subjected to calcination to obtain Ru-doped cobalt oxide rich in oxygen vacancies [53]. Sun et al. [54] prepared a porous NiCo_2_O_4_/NiO hollow dodecahedron using zeolitic imidazolate framework-67 as the precursor and self-sacrificing template. Specifically, the prepared ZIF-67 powder was placed in anhydrous ethanol solution with dissolved nickel nitrate, and then the target product was obtained through heating, centrifugation, washing, drying, and calcination.

### 2.3. Chemical Reaction

In addition to the thermal transformation pathway, MOF materials can also be transformed to metal oxides by chemical solution reactions at room temperature. Zhang and other researchers [55] synthesized the Prussian blue analog (PBA) Mn_3_[Co(CN)_6_]_2_·9H_2_O by reacting polyvinyl pyrrolidone (PVP), Mn(CH_3_COO)_2_·4H_2_O, and K_3_[Co(CN)_6_]. They further successfully prepared Mn_3_O_4_ nanostructured materials using a room-temperature alkaline treatment method. The specific experimental process was as follows: Mn_3_[Co(CN)_6_]_2_·9H_2_O nanocubes were dispersed in 200 mL of NaOH solution with a specific concentration. After stirring for 5 min, the product was collected, washed with anhydrous ethanol and deionized water, and then dried at 80 °C. The study revealed that the concentration of NaOH has a significant regulatory effect on the morphology of the product: under low-concentration conditions, yolk–shell structured Mn_3_O_4_ can be obtained, while under high-concentration conditions, the cubic structure is completely dissociated, forming a flower-like structure assembled from ultrathin nanosheets. Abney et al. [56] used NaOH treatment of MIL-125 and UiO-66 MOF to prepare TiO_x_ and ZrO_x_.

## 3. Application of MOF-Derived Metal Oxides in SCR Reactions

### 3.1. MIL-Derived Metal Oxides

Materials of Institute Lavoisier frameworks (MIL) are a highly representative class of MOFs, widely recognized for their high surface area, tunable pore structures, and excellent stability [57]. These materials were first synthesized by Ferey’s team at the Lavoisier Institute of Materials through the hydrothermal treatment of terephthalic acid and chromium nitrate, and were named “MIL” after the institute’s abbreviation. The developed MIL series mainly includes typical structures such as MIL-53, MIL-100, and MIL-101. MIL-53 has the molecular formula M^III^(OH) and the hydrated form [O_2_C-C_6_H_4_-CO_2_]·H_2_O (M^III^ = Al, Cr, Fe) and is characterized by a flexible three-dimensional structure and a one-dimensional array of macroporous channels. The framework of MIL-100 consists of three octahedra sharing a μ3-O common vertex interconnected by 1,3,5-benzene tricarboxylate (BTC) ligands. MIL-101 has a zeolite-like structure consisting of 1,4-benzenedicarboxylate (1, 4-BDC) bound to a similar trimer. In recent years, the application of derivatives from Institut Lavoisier (MIL) MOFs in the SCR process for NO_x_ degradation has achieved outstanding progress (Table 2).

Qin et al. [58] prepared FeO_x_/C by heat-treating the MIL-101(Fe) precursor, and controlled the rapid growth of the nanocrystals by using CO as a crystal facet guide during the heat treatment. It was found that the catalyst with more exposed (111) facets had stronger denitrification performance compared to the material with exposed (100) facets, with a conversion of 82.3% at 300 °C, while the former only reached 42.3% at the same temperature. The Fe_3_O_4_ (111) catalysts were also highly water-resistant, and the catalytic activity was essentially unaffected by the presence of 10 vol.% H_2_O. The preferential exposure of Fe_3_O_4_ (111) crystalline surfaces increased the concentration of adsorbed oxygen on the catalysts, showed higher surface acidity, and enhanced the interaction between NO, O_2_, and the catalysts. Hu et al. [59] prepared Fe-MIL-101-NH_2_ by a solvothermal method using iron nitrate (FeNO_3_⋅6H_2_O) and 2-aminoterephthalic acid (DBCNH_2_) as raw materials and then calcined this precursor to obtain octahedral α-Fe_2_O_3_ catalysts. The optimal synthesis conditions for the catalyst were obtained by adjusting the calcination temperature and time. The results showed that the NO conversion rate of the catalyst calcined at 400 °C for 1 h was the highest, reaching 90.2% at 350 °C, which is about 40% higher than that of α-Fe_2_O_3_(HT) prepared by the traditional hydrothermal method. The excellent activity of the derived catalyst is attributed to the formation of new pores, which create a greater number of Lewis acid sites and, thereby, improve the conversion efficiency of NO_x_. Zhang et al. [60] prepared MnO_x_-Fe_3_O_4_ nanomaterials with excellent CO-SCR activity by pyrolysis of Mn-MIL-53(Fe) in an inert atmosphere. The material with the optimal metal ratio achieved 97.5% NO_x_ conversion. Characterization results showed that the Mn species effectively promoted the reaction by reducing the grain size, enhancing the reducibility, boosting the mobility, and increasing the amount of lattice oxygen. Jiang et al. [61] loaded phosphotungstic acid (HPW) onto MIL-100(Fe) via three methods: hydrothermal (HT), impregnation (IM), and grinding (GR), and then pyrolyzed the mixed precursors in a N_2_ atmosphere to prepare composite Fe-based NH_3_-SCR catalysts. Among them, the HPW-FeO_x_(MOFs)-HT catalyst prepared by the hydrothermal method retained the super-tetrahedral structure of the MOFs precursor and exhibited the best activity, achieving over 90% NO_x_ conversion across a wide temperature range of 205–470 °C. The presence of 5 vol.% H_2_O only led to a three-percentage-point decrease in catalytic activity. The hydrothermal method was found to promote a favorable interaction between W and Fe, resulting in the formation of γ-Fe_2_O_3_ and an elevation in active oxygen species. This, in turn, bolstered the adsorption and activation of NO, thus speeding up the SCR reaction rate. Moreover, HPW not only augmented the total number of acidic sites but also enhanced the reactivity of NH_3_ adsorbed at Lewis acid sites at low temperatures.

**Figure 3:**
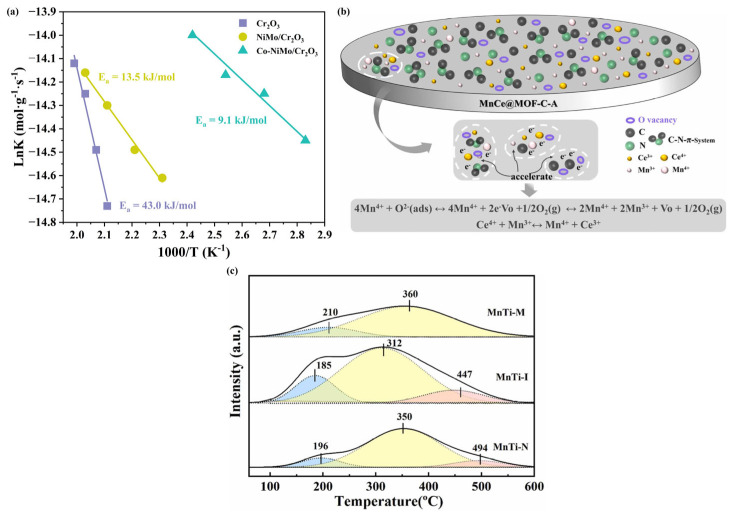
(**a**) Arrhenius plots of catalysts [63]; (**b**) electron transfer on the surface of catalyst MnCe@MOF-C-A [66]; (**c**) the NH_3_-TPD profiles of catalysts [65].

Yu et al. [62] synthesized novel CrO_x_/C nanoparticles for low-temperature NH_3_-SCR reaction using an MOF (MIL-101(Cr))-assisted process. It was found that the MOF-derived CrO_x_/C catalysts possessed higher activity compared to the Cr_2_O_3_/C-WI catalysts prepared using wet impregnation. A series of characterization analyses of the catalysts confirmed that the CrO_x_/C catalysts were mainly composed of CrO_x_ nanoparticles with Eskolaite phase structure as well as activated lattice oxygen. It was hypothesized that the presence of activated lattice oxygen was the key factor for the significant enhancement of NH_3_-SCR activity of the CrO_x_/C catalyst. Whereas the Eskolaite phase structure endowed CrO_x_ with stable lattice properties, this stability effectively suppressed the occurrence of the sulfation reaction, which in turn provided the catalyst with good SO_2_ tolerance and excellent regeneration capability. Our research team prepared a uniform porous Co-NiMo/Cr_2_O_3_ composite material using POMs@MIL-101(Cr) (POMs = polyoxometalates) core–shell templates as precursors. The prepared Co-NiMo/Cr_2_O_3_ catalyst exhibited excellent NO_x_ reduction activity, with a NO_x_ conversion of 95% at 150 °C and over 87% in the temperature range of 150–300 °C. The outstanding activity is attributed to the uniform dispersion of the Co-NiMo/Cr_2_O_3_ catalyst without aggregation, as well as its abundant exposed active sites, large specific surface area, rich lattice oxygen, and acidic sites. Kinetic analysis reveals that the Co-NiMo/Cr_2_O_3_ catalyst’s lower activation energy enables easier reactant activation, consistent with its superior catalytic performance (Figure 3a) [63]. Zhu et al. [64] reported a study on the application of a carbon-containing derivative material made from MIL-101-Cr as a precursor and MnO_x_-CeO_x_ as an active component in NH_3_-SCR. The derivatives can achieve 100% NO_x_ conversion in the temperature range of 150–225 °C. The remarkable activity of the catalyst is mainly attributed to the following factors: firstly, the residual carbon has good electrical conductivity, which can effectively promote the electron transfer between the active components and, thus, significantly improve the catalytic performance; secondly, the residual carbon has reducing properties, which can promote the generation of oxygen vacancies, facilitating the smooth progress of redox reactions. In addition, the presence of isolated electrons in the residual carbon acts like an electron supply station, balancing the electron transfer process or increasing the local charge density, thereby further enhancing the catalyst’s performance. The team also selected MIL-125-Ti, which has a regular pill morphology, a large specific surface area, and good thermal stability, as the precursor, and loaded the active components Mn/Ce on it by wet impregnation. Subsequently, after calcining the MOF precursor loaded with active components in different oxygen concentration atmospheres (pure oxygen, air, nitrogen), a series of nanocomposite materials were obtained. The experimental results showed that the catalyst calcined in air had the best denitrification performance, as well as good H_2_O and SO_2_ resistance. Further research indicated that this may be mainly attributed to the unique porous structure composed of well-dispersed MnO_x_-CeO_x_ and N-doped carbon framework, which can effectively increase the electrical conductivity to accelerate the transfer of electrons (Figure 3b). In addition, the appropriate oxygen content in the calcination atmosphere not only increased the specific surface area of the catalyst but also generated more oxygen vacancies on the catalyst surface [66]. Du et al. [65] successfully synthesized MIL-125(Ti) material via a solvothermal method, followed by an in situ doping strategy to incorporate Mn into the MOF. After high-temperature calcination, a unique spindle-shaped Mn-Ti composite catalyst was obtained. The study revealed that the high surface area and framework advantages of the MOFs precursor enabled highly dispersed active sites in the MnTi-I catalyst, with the in situ derivation process further enhancing this characteristic. Characterization results demonstrated that the catalyst surface exhibited an optimized Mn^4+^/Mn^3+^ oxidation state ratio, abundant chemically adsorbed oxygen species, and the highest number of acid sites (Figure 3c). These combined properties synergistically contributed to its outstanding catalytic performance in high-temperature NH_3_-SCR reactions.

### 3.2. ZIF-Derived Metal Oxides

Zeolitic imidazolate frameworks (ZIFs) are a class of MOFs first reported in 2002. They consist of transition divalent metal ions (such as Zn or Co) connected with imidazolate-derived organic linkers, forming a molecular sieve framework with a zeolite-like structure [67,68]. Structurally, ZIF materials are constructed from M(Imi)_4_ (where Imi represents imidazolate) tetrahedral units, which are interconnected through coordination between divalent metal cations and imidazolate anions. Here, the imidazolate groups act as “bridges” between metal nodes. Notably, the metal ions play a role analogous to silicon atoms in the structure, while the connecting bonds formed by imidazolate anions function similarly to oxygen atoms in zeolites. Due to the M-Im-M bond angle (approximately 145°) formed between imidazolate and transition metals being close to the typical Si-O-Si bond angle in zeolites, ZIFs can replicate various zeolitic topological frameworks [69]. Although zinc ions (Zn^2+^, as in ZIF-8) and cobalt ions (Co^2+^, as in ZIF-67) are the most common metal centers, the structure also allows for substitution with other divalent metal ions such as copper (Cu^2+^), nickel (Ni^2+^), and cadmium (Cd^2+^), thereby endowing the material with tunable properties [70]. Due to their chemical and thermal stability, high porosity and crystallinity, ease of synthesis, and environmental friendliness, ZIFs have attracted widespread attention [71]. ZIF derivatives possess highly stable porous structures and have been extensively studied and applied in the field of catalysis.

**Figure 4:**
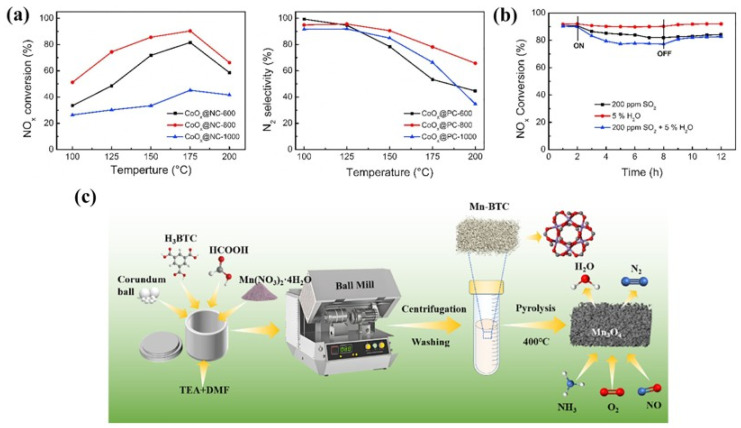
(**a**) NH_3_-SCR activity and N_2_ selectivity of catalyst [72]; (**b**) SO_2_ and H_2_O resistance test [72]; (**c**) schematic diagram of MnO_x_ preparation by mechanical method [75].

Bai et al. [72] prepared a series of Co_3_O_4_ nanoparticles embedded in porous carbon (CoO_x_@PC-T) through a two-step strategy of first pyrolyzing ZIF-67 under an Ar atmosphere and then oxidizing with O_2_. The results showed that the pyrolysis temperature had a significant effect on the carbonization degree of carbon in ZIF-67, which in turn affected the exposure and oxidation states of cobalt nanoparticles on the catalyst surface. Among the series of catalysts prepared, CoO_x_@PC-800 exhibited the highest NO_x_ conversion rate, achieving a maximum conversion of 90.4% at 175 °C (Figure 4a). The catalyst also showed 90% activity retention after 72 h of continuous operation, indicating good stability and promising practical utility. This excellent performance was mainly attributed to its higher Co^3+^/Co^2+^ ratio and larger specific surface area, both of which endowed CoO_x_@PC-800 with outstanding performance in NO_x_ conversion reactions. In addition, when 200 ppm SO_2_ was present in the reaction system, the catalyst’s activity decreased by only 8%. And when 5 vol.% H_2_O was present, the activity decreased by only 2%, demonstrating the catalyst’s excellent sulfur and water resistance (Figure 4b). Our research team [73] prepared MnW/Co_3_O_4_ composite materials with porous structures and highly dispersed active components by pyrolyzing Mn-ZIF encapsulated polyoxometalate precursors. This material can achieve 100% NO_x_ conversion at 250 °C. Its excellent performance is mainly attributed to the following aspects: (1) The active components are uniformly distributed, which provides a good basis for efficient catalytic reactions. (2) The synergistic effect between Mn and Co establishes an effective redox cycle (Mn^4+^ + Co^2+^ → Mn^3+^ + Co^3+^; Mn^2+^ + Co^3+^ → Mn^3+^ + Co^2+^). (3) The generation of oxygen vacancies and acid sites. (4) The enhanced adsorption and activation properties of NH_3_ and NO. Kim and his research team successfully synthesized two-dimensional sheet-like porous anatase titanium dioxide (TiO_2_(Z)) with a high specific surface area using ZIF-8 as a sacrificial template. Subsequently, MnO_x_ was loaded onto this support via wet impregnation, and the catalytic performance of Mn/TiO_2_(Z) was systematically investigated and compared with MnO_x_ catalysts supported on commercial anatase TiO_2_ (TiO_2_(G) and TiO_2_(H)). The experimental results demonstrated that Mn/TiO_2_(Z) exhibited significantly superior NH_3_-SCR performance compared to the commercial support-based catalysts: at a reaction temperature of 150 °C, its NO_x_ conversion reached 96%, while Mn/TiO_2_(G) and Mn/TiO_2_(H) achieved only 54% and 62%, respectively. Further analysis revealed that MnO_x_ was primarily distributed within the micropores of the commercial supports TiO_2_(G) and TiO_2_(H), whereas the unique high external surface area of TiO_2_(Z) facilitated the enrichment of MnO_x_ in the form of clusters on the outer surface of the support. In situ DRIFTS characterization revealed that the formation of bridged nitrate species played a critical role in the catalytic activity during low-temperature NH_3_-SCR reactions, and these active species were more likely to form on MnO_x_ clusters rather than on highly dispersed MnO_x_. Due to its larger external surface area, TiO_2_(Z) promoted the formation of MnO_x_ clusters on the outer surface, thereby significantly enhancing the low-temperature SCR activity of the catalyst [74].

### 3.3. BTC Series MOF-Derived Metal Oxides

Cu-BTC, as a prototypical representative of the BTC series of metal–organic frameworks, was first synthesized and reported by Chui’s research team in 1999. This material features a distinctive paddle-wheel geometric structure, where each structural unit consists of two Cu^2+^ ions coordinated with four carboxylate groups, ultimately forming a three-dimensional porous network with the chemical formula [Cu_3_(BTC)_3_(H_2_O)_3_]_n_ [76]. Through a metal ion substitution strategy, the copper metal centers in Cu-BTC can be replaced with other transition metal elements such as Mn, Ce, and Co, yielding derivatives like Mn-BTC, Ce-BTC, and Co-BTC [77,78,79]. The metal oxides derived from these BTC-based MOF materials via pyrolysis demonstrate significant application value in catalytic denitrification due to their unique pore structures and abundant active sites.

The research team of Jia et al. found that the CuO_x_/C catalyst obtained by calcining the Cu-BTC precursor at 450 °C for 3 h has good denitrification performance, with a denitrification rate of about 80% at 240 °C and over 90% at 300 °C. Material characterization results show that the CuO_x_/C catalyst contains crystalline structures of CuO and Cu_2_O. As the calcination temperature increases, the crystalline structure of Cu_2_O gradually decreases. Moreover, the coexistence of appropriate amounts of Cu^+^ and Cu^2+^ can form electron pairs, accelerate the redox cycle, and thereby enhance the denitrification activity of the catalyst. When the temperature of the SCR reaction is 100 °C, the side reactions are relatively complex, producing a variety of by-products, including N_2_O, NO_2_, N_2_O_3_, N_2_O_4_, and N_2_O_5_. However, when the temperature rises to 300 °C, due to the increase in the Gibbs free energy of N_2_O_3_, N_2_O_4_, and N_2_O_5_, these reactions cannot proceed spontaneously, and only N_2_O and NO_2_ remain as by-products in the system [80]. Zhang et al. [81] also demonstrated that the activity of CuO_x_/C catalysts prepared from Cu-BTC precursors was significantly better than that of single CuO. Qin et al. [82] thermally treated mixed node A-Cu-BTC (A = Sr, La, Ce, Al) under N_2_ atmosphere. It was found that SrO_x_/CuO_y_/C could reach 100% CO-SCR activity at 172 °C, while the reaction temperature to obtain the same conversion for single CuO_y_/C increased to 265 °C. A (Sr, La, Ce, Al) atoms may be carriers of electrons, and the addition of A atoms helps to reduce CuO to Cu^+^ cations. Shi’s research team conducted a comprehensive investigation into the performance of Fe-, Co-, and Ni-doped Cu-BTC bimetallic MOF-derived catalysts in the CO-SCR reaction. The experimental results demonstrated that these bimetallic catalysts exhibited significantly enhanced catalytic activity and N_2_ selectivity in the low-temperature range of 100–350 °C compared to the monometallic Cu/TiO_2_ catalyst, with Fe-Cu/TiO_2_ showing the best performance, followed by Co-Cu/TiO_2_, while Ni-Cu/TiO_2_ was relatively less effective. Specifically, Fe-Cu/TiO_2_ achieved nearly complete NO conversion over a broad temperature window of 150–350 °C. This outstanding catalytic performance stemmed from its unique physicochemical properties. On one hand, the catalyst exhibited remarkable redox capability, with a surface-active oxygen species ratio (O_β_/(O_α_ + O_β_)) as high as 32.90% and an Fe^2+^/Fe^3+^ ratio of 1.79. On the other hand, in situ FTIR analysis revealed that Fe doping not only facilitated the formation and stabilization of nitrate species but also significantly increased the number of Lewis acid sites via the formation of Cu^+^-CO intermediates, thereby substantially improving catalytic efficiency [83].

**Figure 5:**
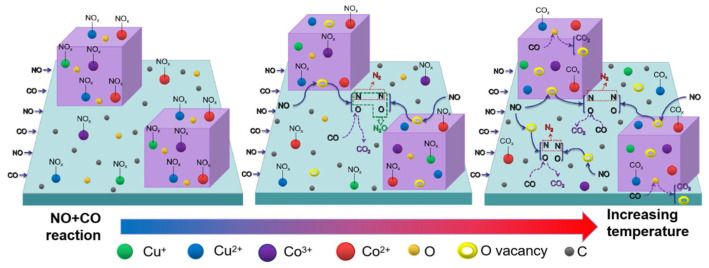
The mechanism illustration of Co_0.75_-CuO_x_/C for NO + CO reaction [85].

Li et al. [75] synthesized a series of amorphous derivatives of MnBTC-BM MOF via a mechanochemical method and applied them to the NH_3_-SCR reaction. The detailed preparation process is illustrated in Figure 4c. The Mn (II, III) oxide obtained after thermal decomposition at 400 °C retained its amorphous characteristics. Compared with derivatives prepared via hydrothermal methods and at other temperatures, it exhibited a smaller crystallite size and lower Mn-O bond energy. This resulted in an increase in surface defects and acid sites, facilitated the release of lattice oxygen, and consequently led to optimal low-temperature NH_3_-SCR activity (achieving 90% NO conversion at 170 °C) as well as the lowest apparent activation energy (18 kJ/mol). Ko et al. [84] investigated the relationship between the catalytic performance of Mn, Co-BTC derivatives in the NH_3_ selective catalytic reduction in NO_x_ and the annealing temperature (300–600 °C). The catalytic activity test results indicated that the annealing temperature had a significant promoting effect on the catalytic performance. Mn, Co-BTC-300 exhibited NO_x_ conversion rates of 78–80% at 200–250 °C, with lower catalytic activity at other reaction temperatures. In contrast, Mn, Co-BTC-x (x = 400, 500 °C) achieved NO_x_ conversion of over 90% in the 150–300 °C range. SEM, XRD, and FTIR test results demonstrated that as the annealing temperature increased, Mn, Co-BTC gradually transformed into spinel oxides with a honeycomb structure. XPS and H_2_-TPR results revealed that Mn, Co-BTC-500 had the greatest synergistic electronic interaction between Mn and Co ions, which might be the key factor for its excellent SCR catalytic activity. Zhao et al. [85] used Cu-BTC as a template and employed the impregnation method to load different proportions of Co, followed by calcination to prepare heteroepitaxial metal oxides with a regular morphology, excellent performance, and high stability on a carbonaceous framework. The study found that CuO, Cu_2_O, and Co_3_O_4_ coexisted in multiple phases in the pyrolysis products, with carbon highly dispersed on the surface of the catalyst. Carbon containing lone pairs of electrons can facilitate electron transfer between Cu and Co, thereby enhancing catalytic activity. Among the series of catalysts, Co_0.75_-CuO_x_/C exhibited the best catalytic ability, achieving a denitrification efficiency of over 90% at 175 °C. It was found that at low temperatures below 175 °C, nitric oxide (NO) is primarily converted to nitrous oxide (N_2_O) first, and only with further temperature increase will it be converted to nitrogen (N_2_). The reason is that at lower temperatures, nitrates and nitrites tend to decompose to form nitrous oxide rather than directly generating nitrogen gas, reflecting that relatively fewer N-O bonds are broken in this temperature range. According to in situ DRIFTS studies, the mechanism of the CO-SCR reaction on the Co_0.75_-CuO_x_/C catalyst is as follows (Figure 5): When the reaction temperature is below 200 °C, the transformation of gaseous NO mainly occurs. At room temperature, when the catalyst comes into contact with a mixture of gaseous CO and NO, NO molecules are preferentially adsorbed to form NO_x_ species. As the temperature rises, NO_x_ decomposes, generating oxygen vacancies that weaken the N-O bonds and cause NO to dissociate into [O] and [N] radicals. The [O] radical combines with CO to form CO_2_, while the [N] radical partly combines with NO to form N_2_O and partly combines with itself to form N_2_. When the reaction temperature is between 225 °C and 300 °C, the active oxygen on the catalyst surface is easily captured by CO, forming oxygen vacancies and generating carbonate and carboxylate species with metal oxides. Under high-temperature reducing conditions, NO_x_ species decompose and transform in large quantities, almost completely disappearing, and N_2_O is further reduced to N_2_. Liu et al. [86] successfully synthesized Ce-Ti MOF materials using Ce(NO_3_)_3_·6H_2_O and Ti-(OCH_2_CH_2_CH_2_CH_3_)_4_ as metal precursors, with 1,3,5-benzenetricarboxylic acid (H_3_BTC) as the organic ligand. Subsequently, a series of MOF-derived CeTiO_x_ catalysts were obtained by calcination at different temperatures (300, 400, 500, 600, and 700 °C). The study found that the calcination temperature significantly influenced the NH_3_-SCR performance of the CeTi-T catalysts. Among them, CeTi-400 exhibited the best catalytic performance: within a broad temperature window of 183–422 °C, the NO_x_ conversion remained above 80%, while the N_2_ selectivity approached 100% in the range of 150–450 °C. Additionally, this catalyst demonstrated excellent resistance to SO_2_/H_2_O. The systematic characterization revealed that the CeTi-400 catalysts had more Ce-O-Ti components, which resulted in a larger specific surface area and more acidic sites, as well as a richer Ce^3+^ species and a higher concentration of adsorbed oxygen on the surface, which was conducive to the improvement of the NH_3_-SCR performance.

### 3.4. UiO-Derived Metal Oxides

In the UiO family of metal–organic frameworks (MOFs), the metal center Zr^4+^ together with the dicarboxylic acid linker constitutes their basic structure, covering various members such as UiO-66, UiO-67, and UiO-68 [87]. Despite the differences in ligand lengths used in these subfamily members (UiO-66, UiO-67, and UiO-68), their network topologies show a high degree of similarity. UiO-MOFs possess an expanded cubic close-packed (CCP) structure with Zr(v) cations forming the central nodes [88]. The chemical formulas of UiO-66, UiO-67, and UiO-68 are Zr_6_O_4_(OH)_4_(BDC)_6_, Zr_6_O_4_(OH)_4_(BPDC)_6_, and Zr_6_O_4_(OH)_4_(TPDC)_6_, respectively. The organic linkers used in these compounds are terephthalic acid (BDC, also known as 1,4-benzenedicarboxylic acid), 4,4′-biphenyl dicarboxylic acid (BPDC), and triphenyl dicarboxylic acid (TPDC). The excellent structural properties of UiO-MOFs can be converted into oxide materials with unique physicochemical properties, which exhibit highly efficient NO_x_ reduction in the SCR reaction.

Wang et al. [89] prepared a catalyst consisting of carbon-coated octahedral ZrO_2_ with highly dispersed Pt particles using UiO-66-NH_2_ as a template. The H_2_-SCR performance of the catalysts with different Pt loading ratios was investigated, and it was found that 0.1 wt% Pt/ZrO_2_@C could achieve nearly 100% NO conversion at 90 °C. However, when the Pt content exceeds 0.1 wt%, the catalytic activity decreases. This result is attributed to the large amount of Pt particle agglomeration leading to a decrease in active site exposure, which negatively affects the catalytic activity of the H_2_-SCR reaction. Han et al. [90] successfully prepared a series of Ce-Zr bimetallic MOF derivatives by pre-annealing Ce_x_Zr_1−x_/UiO-66 at different temperatures in an air atmosphere. Among them, the C_0.5_Z_0.5_/U-400 catalyst demonstrated optimal NH_3_-SCR activity, achieving 92% NO_x_ conversion at 350 °C while maintaining 100% N_2_ selectivity over a broad temperature range of 150–500 °C. Through comprehensive characterization techniques, the effects of pre-annealing temperature on the microstructure and chemical properties of C_0.5_Z_0.5_/U materials were systematically investigated. The study revealed the following: (1) Although pre-annealing treatment reduced the specific surface area, derivatives obtained after 2 h annealing at 400 °C retained relatively large surface area and developed a fluffy porous structure, facilitating full exposure of active sites. (2) The C_0.5_Z_0.5_/U-400 catalyst exhibited three key advantageous features: a high Ce^3+^/(Ce^3+^ + Ce^4+^) ratio, excellent redox capability, and abundant oxygen vacancies. These characteristics synergistically promoted the adsorption and activation of reactant gases, thereby significantly enhancing catalytic performance.

### 3.5. MOF-74- and MOF-5-Derived Metal Oxides

Among various types of MOF materials, MOF-74 has attracted much attention due to its unique structural advantages. The material has a honeycomb hexagonal pore network structure, and the hydroxyl and carboxyl oxygen atoms in its organic ligands provide a stable coordination environment for the metal centers through synergistic coordination. This special structural feature enables MOF-74 to expose a large number of high-density metal active sites, a property that makes it an ideal precursor for the preparation of metal oxide catalysts. Table 3 summarizes the structural characteristics, preparation strategies, and NO_x_ removal performance of MOF-74-derived metal oxides.

**Figure 6:**
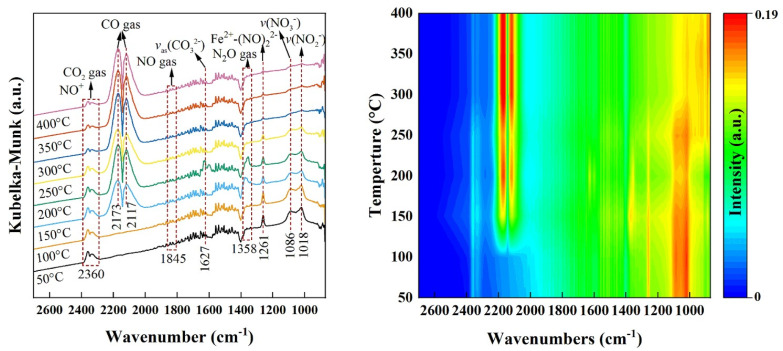
In situ DRIFTS spectra of CO + NO adsorption over Mn_0.5_-Fe_2_O_3_/C catalyst [93].

Zhang et al. [91] used Mn-MOF-74 synthesized by the solvothermal method as a precursor and calcined it under different gas atmospheres (air, 3000 ppm NH_3_, and air mixed with NH_3_) and at various temperatures (300–650 °C) to prepare a series of MnO_x_ nanocomposite catalysts. The results showed that the redox properties of MnO_x_ derivatives could be effectively regulated by adjusting the calcination atmosphere and temperature. Among them, the porous foam-like MnO_x_-350-air + NH_3_ catalyst exhibited excellent catalytic performance, with NO conversion stably maintained at over 80% in the wide temperature range of 150–500 °C and a selectivity for N_2_ reaching 100%. It also possessed good hydrothermal stability. This outstanding low-temperature catalytic activity was mainly attributed to the high Mn^4+^/Mn^n+^ ratio and O_α_/(O_α_ + O_β_) ratio of the material. These active redox sites could promote the adsorption and oxidation of NO, thereby effectively driving the “fast SCR” reaction. Other researchers have synthesized a series of Fe_x_Mn_3−x_O_4_ nanoparticles from the bimetallic Fe-Mn-MOF-74 using a pyrolysis-oxidation strategy. Among them, Fe_0.35_Mn_2.65_O_4_ nanoparticles showed a NO conversion of up to 90% at 180 °C at an ultra-high gas hourly space velocity (GHSV) of 400,000 h^−1^. The catalyst was found to contain an efficient Fe_oct_-O-Mn_tet_ active site with the lowest formation energy of oxygen vacancies, a process that is the rate-determining step in NO oxidation. The high NO-to-NO_2_ oxidation activity triggers a “fast SCR” reaction, which improves the NH_3_-SCR performance [92]. Li et al. [93] similarly prepared a series of spherical Mn-Fe_2_O_3_/C catalysts using MnFe-MOF-74 as a precursor and applied them to the CO-SCR reaction. Among all tested catalysts, Mn_0.5_-Fe_2_O_3_/C catalysts showed excellent performance, with NO conversion up to 100% in a wide temperature interval from 225 to 500 °C, which was much higher than that of monometallic Fe_2_O_3_/C catalysts. With the help of in situ Fourier transform infrared spectroscopy (FT-IR), the researchers identified key Fe^2+^-(NO)_2_^2−^/N_2_O intermediates. These intermediates are effective in lowering the reaction energy barrier and, thus, accelerating the NO reduction reaction (Figure 6). The Chen research team innovatively developed a new process for preparing F-doped Mn_3_O_4_ catalysts based on a three-step method of “crystallization-pyrolysis-oxidation”. This study used Mn-MOF-74 as the precursor and achieved uniform dispersion of fluorine in the cage-like structure of Mn-MOF-74 by introducing NH_4_F as the fluorine source during the hydrothermal synthesis stage. Through the subsequent precisely controlled pyrolysis and oxidation process, the F-modified Mn_3_O_4_ catalyst (F-Mn_3_O_4_-x) was successfully prepared. Performance tests showed that the optimized F-Mn_3_O_4_-3% catalyst could maintain a NO removal efficiency of over 90% in the wide temperature range of 150–310 °C and exhibited better sulfur resistance than undoped Mn_3_O_4_. Mechanistic studies revealed that fluorine doping enhanced the catalyst performance through a dual-action mechanism. On the one hand, the introduction of fluorine effectively regulated the surface acidity of the catalyst, promoting the chemisorption of NH_3_ by increasing acid sites, thereby enhancing the SCR reaction activity. On the other hand, the substitution of lattice oxygen by fluorine atoms significantly altered the electronic structure of the material, inhibiting the adsorption and electron transfer of SO_2_ on the active Mn^3+^ sites. This electronic effect enabled the catalyst to selectively catalyze the oxidation of NO to NO_2_ rather than converting SO_2_ to SO_3_, effectively avoiding catalyst deactivation caused by sulfate deposition [94].

**Figure 7:**
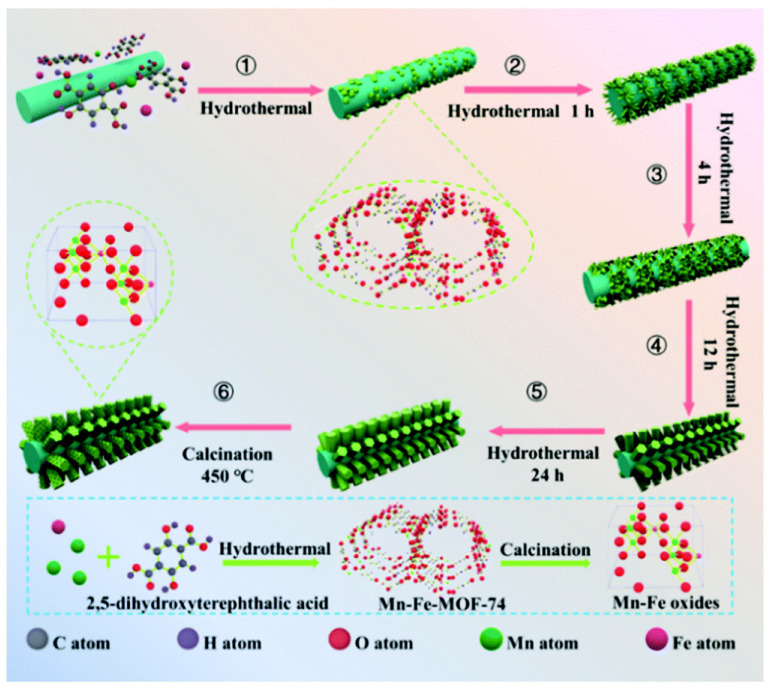
Schematic diagram of the process for the preparation of Mn-Fe mixed oxides [95].

Yao et al. [95] proposed an innovative method for preparing a novel manganese–iron mixed oxide supported on an iron mesh using MOF-74 as the precursor. The preparation process, as shown in Figure 7, mainly includes the following key steps: First, the Fe^3+^ ions in the oxide layer on the surface of the iron mesh act as active sites to induce the nucleation of MOF-74 (step 1). Subsequently, these initial nuclei undergo morphological evolution through a self-assembly process, successively forming needle-like structures (step 2) and sheet-like transitional states (step 3). During the continuous growth phase (step 4), the material ultimately develops into the rod-shaped MnFe-MOF-74 precursor (step 5). Finally, the target Mn-Fe oxide material is obtained through subsequent high-temperature calcination of these precursors. The activity test results showed that the Mn-Fe co-precipitated catalyst could reach up to 86.8% NO conversion, while the Mn-Fe in situ catalyst prepared with MOF-74 as precursor could increase the conversion to 96.6% under the same conditions. The good low-temperature activity of the Mn-Fe in situ catalyst was mainly attributed to the increase in the oxygen defects and the Brønsted acid sites, the significant enhancement of the electron mobility, and the effective generation of key active intermediates during the reaction process. Zhou et al. [96] found that the Cu-MOF-74 derivatives not only retained their original pore structure and specific surface area but also exhibited excellent thermal stability as well as uniformly dispersed active copper species, which together significantly enhanced their activity in the CO-SCR reaction.

As a typical representative of metal–organic framework materials, MOF-5 has a three-dimensional porous structure. Its framework is self-assembled from zinc ion centers and 1,4-benzenedicarboxylate ligands (BDC) through coordination bonds [97]. In recent years, MOF-5 has also been used as a template for the preparation of metal oxides. Zhao’s research team prepared CeO_2_-ZnO composite catalysts by high-temperature pyrolysis using Ce/MOF-5 as the precursor. The study found that the strong interaction between Ce^4+^ and Zn^2+^ significantly enhanced the catalyst’s performance, with the 5% CeO_2_-ZnO catalyst exhibiting the best results, achieving a 69.1% N_2_ yield in the selective catalytic reduction of NO by propylene. Through in situ DRIFTS and NO-TPD analyses, the researchers observed the formation of various key reactive intermediates on the catalyst surface, including monodentate/bidentate nitrates, chelating nitrites, nitro compounds, nitrosyl species, and C_x_H_y_O_z_-type species (such as enolates and acetates). These intermediates were further converted into hydrocarbonate or carbonate species, ultimately decomposing into N_2_, CO_2_, and H_2_O, thereby efficiently completing the catalytic reduction in NO [98].

### 3.6. Prussian Blue Analog-Derived Metal Oxides

Prussian blue analogs (PBAs) are a type of metal–organic frameworks (MOFs) constructed from divalent and trivalent metal ions bridged by cyanide ligands [99]. Due to their considerable specific surface area, good porosity, and unique electronic transfer properties, they exhibit great practical potential. More prominently, based on the unique reactivity and thermal properties of PBAs, they can serve as ideal precursors for the preparation of hierarchical materials with hollow or porous structures.

**Figure 8:**
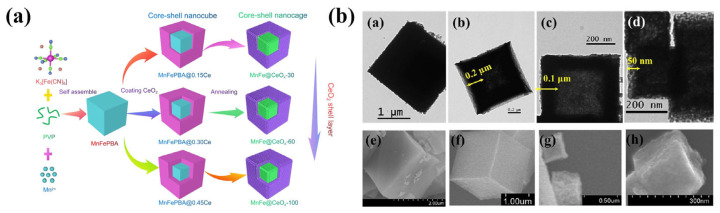
(**a**) Diagram of the synthesis process of catalysts [102]; (**b**) TEM and SEM images of the nanocages with varied scales [105].

Wu et al. [100] successfully constructed a MnCoO_x_@TiO_2_ catalyst with a unique double-walled nanocage structure by thermally treating the self-assembled Mn_3_[Co(CN)_6_]_2_·nH_2_O@Ti(OH)_4_ precursor. This catalyst exhibits excellent low-temperature NH_3_-SCR performance and N_2_ selectivity, with NO conversion efficiency exceeding 90% in the temperature range of 200–325 °C and N_2_ selectivity exceeding 97.5% in the range of 50–250 °C. These phenomena can be explained as follows: The TiO_2_ shell can inhibit the oxidation of NH_3_ in the SCR reaction, and the nanoscale confinement effect of the TiO_2_ shell and the partial substitution of metal oxides due to electrochemical substitution reactions can promote low-temperature activity, selectivity, and stability. Moreover, the interaction between the MnCoO_x_ core and the TiO_2_ shell significantly reduces the number of surface basic sites, thereby suppressing the adsorption of SO_2_ and the formation of inert metal sulfates, maintaining the catalyst’s excellent sulfur resistance. Cai et al. [101] successfully synthesized MnFeO_x_@TiO_2_ double-walled nanocages with a hollow porous structure by a similar approach, and it was shown that the construction of TiO_2_ shells could significantly enhance the adsorption and activation of reactants by the materials. The research group further replaced the TiO_2_ shell with a CeO_2_ shell. The schematic diagram of the catalyst synthesis is shown in Figure 8a. The effect of the CeO_2_ shell thickness on the denitrification performance was systematically investigated. It was found that the MnFe@CeOx-60 catalyst with a shell thickness of 60 nm exhibited the best NH_3_-SCR catalytic activity, with NO removal efficiency exceeding 80% in the temperature range of 120–250 °C. However, an excessively thick CeO_2_ shell partially obscured the surface active sites, thereby inhibiting the catalytic performance. A series of characterizations clearly revealed the structure of the CeO_2_ shell. Due to the diffusion effect at the core–shell interface, some active species diffused across the interface. This interfacial diffusion effect has a dual role: (1) promoting the formation of oxygen vacancy defects and acidic sites, enhancing the adsorption and activation ability of reactants; (2) strengthening the electron transfer between FeO_x_, MnO_x_ and CeO_x_, generating more high-activity species (Mn^4+^, Fe^3+^, Ce^3+^, and O_ads_), thereby improving the efficiency of NO oxidation to NO_2_ and accelerating the rapid SCR reaction process [102]. The research group has also developed a yolk–shell structured MnFe@CeO_x_@TiO_x_ nanocage catalyst. Studies have shown that the CeO_2_ shell can effectively introduce more oxygen vacancy defects, while the TiO_2_ shell can significantly enhance the surface acidic sites. Among them, the MnFe@CeO_x_@TiO_x_-40 catalyst exhibits a catalytic efficiency of over 90% within the broad temperature window of 120–240 °C and maintains a hydrothermal stability of over 90% at 240 °C. This dual-shell design has two major advantages: First, it increases the concentration of high-valent metal ions (Mn^4+^, Fe^3+^, Ce^3+^) and adsorbed oxygen (O_ads_) and strengthens the interfacial synergistic effect between the shells. Second, it cleverly balances the redox properties and surface acidity of the catalyst, thereby significantly enhancing the overall catalytic performance [103].

A research team from Shanghai University has successfully developed hollow and porous Mn_x_Co_3−x_O_4_ nanocages with a spinel structure, which serve as high-performance denitrification catalysts. These nanocages are prepared through the pyrolysis of nanocubic metal–organic frameworks (Mn_3_[Co(CN)_6_]_2_·nH_2_O). The entire fabrication process consists of two main steps. In the first step, Mn_3_[Co(CN)_6_]_2_·nH_2_O nanocubic precursors with well-defined crystal structures are synthesized through shape-controlled methods. Polyvinylpyrrolidone (PVP) plays a crucial role in this process as an efficient capping agent. By preferentially adsorbing onto specific crystal facets, PVP guides the anisotropic growth of the metal cyanide coordination polymer. This preferential capping effect of PVP promotes the self-assembly of Mn^2+^ and [Co(CN)_6_]^3-^ into cubic-shaped nanocrystals. After an Ostwald ripening process, the size of the nanocrystals increases and becomes more uniform. Meanwhile, the crystallinity of the Mn_3_[Co(CN)_6_]_2_·nH_2_O precursor is further enhanced, and structural defects are reduced. In the second step, based on the Kirkendall effect, the Mn_3_[Co(CN)_6_]_2_·nH_2_O precursor nanocubes are annealed to form hollow and porous Mn_x_Co_3−x_O_4_ nanocages. During the annealing process in air, various organic and inorganic components in the prepared Mn_3_[Co(CN)_6_]_2_·nH_2_O precursors, including PVP, crystalline water, and cyanide ligands, decompose and volatilize, ultimately creating the hollow and porous structures. Compared with traditional Mn_x_Co_3−x_O_4_ nanoparticles, the Mn_x_Co_3−x_O_4_ nanocages exhibit superior catalytic activity, higher N_2_ selectivity, a broader operating temperature window, higher stability, and better SO_2_ tolerance in the low-temperature range. Specifically, the reaction temperature required for the nanocages to achieve a 50% conversion rate is only 85 °C, while traditional nanoparticles need a temperature of over 130 °C to reach the same conversion rate. The unique advantage of the hollow and porous structure lies in its ability to provide a larger surface area and more active sites for the adsorption and activation of reactant gases, thereby significantly enhancing catalytic activity. Moreover, the uniform distribution of manganese and cobalt oxides within the nanocages and their strong interactions not only improve the efficiency of the catalytic cycle but also effectively inhibit the formation of manganese sulfate, resulting in higher catalytic cycle stability and good SO_2_ tolerance [104]. The research team also synthesized MnO_x_-FeO_y_ nanocage structures with various particle sizes (0.25, 0.5, 1, and 2 μm) using Prussian blue analogs (Figure 8b). The study found that the 0.5 μm MnO_x_-FeO_y_ nanocage not only exhibited a significant Mn-Fe cation synergistic effect but also demonstrated excellent redox properties. These advantages collectively contributed to a significant improvement in NO_x_ reduction efficiency [105].

## 4. Conclusions and Future Perspectives

This review summarizes the application of MOF-derived oxides in the selective catalytic reduction of NO_x_. Firstly, the preparation strategies of MOF-derived materials are introduced. Secondly, the catalytic activities of representative MOF derivatives and their conformational relationships are summarized in detail. Although significant progress has been made in NO_x_ catalytic reduction using MOF-derived materials, several critical challenges remain to be addressed for their practical applications in the future.

(1) There is an urgent need to strengthen research on the synthesis of MOF-derived materials. Developing highly active derivatives through optimized preparation processes and thoroughly investigating the structure–activity relationships between catalyst microstructure and catalytic performance are essential. Given the direct influence of synthesis methods on the interactions among active components, rational synthesis strategies should be developed to precisely regulate the structural characteristics of catalysts, thereby promoting the industrial application of MOF materials in denitrification. Additionally, the relatively low yield of MOF-derived metal oxides limits their large-scale production. Therefore, exploring novel preparation methods to improve product yield is of great significance for enhancing material synthesis efficiency.
(2) The industrial application of MOF-derived metal oxide denitrification catalysts still faces challenges in balancing cost-effectiveness. The use of metal precursors with high crustal abundance and low price is a key factor to achieve the large-scale production of such catalysts.
(3) Elucidating the catalytic reaction mechanism and revealing the synergistic effect of different elements are the keys to developing efficient and highly selective catalysts. The real-time tracking of reaction dynamics with the help of in situ characterization technology will provide an important basis for the mechanism study.
(4) Future research should pay attention to the application value of density functional theory (DFT) calculations. This method can not only predict the catalyst activity and reduce the cost of experimental screening but also reveal the essence of the catalytic process from the theoretical level and realize the in-depth integration of experimental phenomena and theoretical mechanisms. At the same time, artificial intelligence and machine learning technologies show breakthrough application prospects in the field of material screening and structure design.

## Figures and Tables

**Table 1:** Comparison of preparation methods for metal oxides derived from MOFs.

Preparation Method	Typical Examples	Reaction Condition	Structural Feature	Advantage	Ref.
Self-pyrolysis	(Ni, Cu)O@C	air 400 °C 3 h	hierarchical porous composite with macropores	simple synthesis method, easy to operate, capable of precise control over the composition and morphology of the resulting oxides	[42]
	FeMnO@Sn	air 500 °C 6 h	heterostructure, high specific surface area		[43]
	MgO/C	N_2_ 400 °C 3 h	nanosheets hierarchical nanopore structures		[44]
	Mn_2_O_3_-Fe_3_O_4_	air 500 °C 2 h	hollow porous nanocube structure, low density, maintain fragment uniformity and porosity		[45]
	FeCoNi oxides	air 500 °C 3 h	ternary phase composite, hexagonal rod-like morphology, mesopores		[46]
	MnCeO_x_	Ar for 3 h, followed by air for 3 h	strong intermetallic interactions, high specific surface area, small-sized nanoparticles		[47]
	Pd@ZrO_2_	Ar at 600 °C for 3 h, followed 30 vol.% O_2_/Ar for another 30, 60, 120, and 180 min	large pore size, excellent reducibility, abundant oxygen vacancies		[48]
Loading self-pyrolysis	Cu_x_-O-Ni_y_-O-Co_3−x−y_	calcination temperature (300, 350, 400, and 450 °C), reaction time (1, 2, 4, and 6 h)	dodecahedral shape, hollow	enhanced structural stability, precise morphology control, tailored composition and interfacescomposition and interfaces, extended functionality	[49]
	CeO_2−x_-MnO_x_	air 400 °C 20 min	small grain size, cubic-like structure, rough and porous		[50]
	MnO/Cu-C	N_2_ 600 °C 4 h	nanotube-like morphology		[51]
	CoCeOx	air 500 °C 4 h	octahedral structure, high specific surface area		[52]
	ZIF-Co_3_O_4_-Ru	air 300 °C 2 h	hydrophilicity, nanosheets		[53]
	NiCo_2_O_4_/NiO	air 400 °C 1 h	hollow dodecahedron, polycrystallinity		[54]
Chemical reaction	δ-MnO_2_	NaOH room temperature	nanoboxes, hierarchical pore size, large surface area	low-temperature synthesis and energy conservation, pore size, structure, and composition of the material can be regulated by selecting the treatment solution	[55]
	TiO_x_, ZrO_x_	NaOH oscillation	mesopores, retain the volumetric surface area of the MOF precursor		[56]

**Table 2:** The NO_x_ conversion efficiency of MIL-derived metal oxides.

MOF Precursor	Derived Metal Oxides	Reaction Conditions	T_max_ (°C)	NO_x_ Conversion	Ref.
MIL-101 (Fe)	FeO_x_/C	[NH_3_] = [NO] = 500 ppm, [O_2_] = 5 vol.%, N_2_ balance, GHSV = 30,000 h^−1^	300	82.3%	[58]
Fe-MIL-101-NH_2_	α-Fe_2_O_3_	[NH_3_] = [NO] = 500 ppm, [O_2_] = 3 vol.%, Ar balance, GHSV = 36,000 h^−1^	350	90.2%	[59]
Mn-MIL-53 (Fe)	MnO_x_-Fe_3_O_4_	[NO] = 1 vol.%, [CO] = 2 vol.%, Ar balance	500	97.5%	[60]
HPW-MIL-100 (Fe)	HPW-FeO_x_	[NH_3_] = [NO] = 500 ppm, [O_2_] = 5 vol.%, Ar balance, GHSV = 50,000 h^−1^	205	over 90%	[61]
MIL-101 (Cr)	CrO_x_/C	[NH_3_] = [NO] = 500 ppm, [O_2_] = 5 vol.%, N_2_ balance, GHSV = 30,000 h^−1^	150	over 90%	[62]
POMs@MIL-101 (Cr)	Co-NiMo/Cr_2_O_3_	[NH_3_] = [NO] = 500 ppm, [O_2_] = 5 vol.%, N_2_ balance, GHSV = 60,000 h^−1^	150	95%	[63]
MnCe@MOF-C	MnO_x_-CeO_x_	[NH_3_] = [NO] = 500 ppm, [O_2_] = 5 vol.%	150	100%	[64]
MnTi-MOFs	MnTi	[NH_3_] = [NO] = 500 ppm, [O_2_] = 5 vol.%, N_2_ balance, GHSV = 36,000 h^−1^	150	97%	[65]

**Table 3:** Summary of structure, preparation strategies, and NO_x_ conversion efficiency of MOF-74-derived metal oxides.

MOF Precursor	Derived Metal Oxides	Structures	Strategies	Reaction Conditions	T (°C)	NO_x_ Conversion	Ref.
Mn-MOF-74	MnO_x_	foam-like	calcination in various gas atmospheres (air, 3000 ppm NH_3_, air + NH_3_)	[NH_3_] = [NO] = 1000 ppm, [O_2_] = 7 vol.%, N_2_ balance, GHSV = 7200 h^−1^	223–445	over 90%	[91]
Fe-Mn-MOF-74	Fe_x_Mn_3-x_O_4_	nanoparticles	pyrolysis-oxidation	[NH_3_] = [NO] = 600 ppm, [O_2_] = 5 vol.%, N_2_ balance, GHSV = 400,000 h^−1^	180	over 90%	[92]
MnFe-MOF-74	Mn-Fe_2_O_3_/C	spherical	pyrolysis	[NO] = 500 ppm, [CO] = 1000 ppm, Ar balance, GHSV = 30,000 h^−1^	225–500	100%	[93]
F-Mn-MOF-74	F-Mn_3_O_4_-3%	hexagonal rod structures	crystallization-pyrolysis-oxidation	[NH_3_] = [NO] = 600 ppm, [O_2_] = 5 vol.%, N_2_ balance, GHSV = 400,000 h^−1^	150–310	over 90%	[94]
MnFe-MOF-74	Mn–Fe oxides	rod polyhedral structure	calcination	[NH_3_] = [NO] = 500 ppm, [O_2_] = 5 vol.%, N_2_ balance, GHSV = 10,000 h^−1^	150–210	over 90%	[95]
Cu-MOF-74	Cu/C	rod-like	calcination	[NO] = 500 ppm, [CO] = 1000 ppm, [O_2_] = 1 vol.%, N_2_ balance, GHSV = 30,000 h^−1^	350–500	100%	[96]
